# Determinants for Perinatal Mortality in South China: A Prospective Cohort Study

**DOI:** 10.3389/fped.2022.756444

**Published:** 2022-03-18

**Authors:** Yiping Liu, Qiongxuan Li, Tingting Wang, Senmao Zhang, Letao Chen, Yihuan Li, Jingyi Diao, Jinqi Li, Xinli Song, Mengting Sun, Jianhui Wei, Jing Shu, Tubao Yang, Jiabi Qin

**Affiliations:** ^1^Department of Epidemiology and Health Statistics, Xiangya School of Public Health, Central South University, Changsha, China; ^2^National Health Committee (NHC) Key Laboratory of Birth Defect for Research and Prevention, Hunan Provincial Maternal and Child Health Care Hospital, Changsha, China; ^3^Hunan Provincial Key Laboratory of Clinical Epidemiology, Changsha, China; ^4^Guangdong Cardiovascular Institute, Guangdong Provincial People’s Hospital, Guangdong Academy of Medical Sciences, Guangzhou, China

**Keywords:** perinatal death, perinatal mortality, prospective cohort study, Poisson regression, determinants

## Abstract

**Objective:**

To estimate the association of selected maternal and fetal characteristics with the risk of perinatal mortality in South China.

**Methods:**

A prospective cohort study was conducted from March 2013 to December 2019. The exposures of interest were maternal sociodemographic characteristics, lifestyle and habits during early pregnancy, and complications of pregnancy. Their effects on the development of perinatal death were analyzed in our study.

**Results:**

A total of 44,048 eligible pregnant women were included in the analysis. Of these, 596 fetuses were perinatal deaths (perinatal mortality was 13.5 per 1,000 births). After adjustment, maternal obesity, being employed, history of gestational hypertension, taking antidepressants during early pregnancy, history of gestational diabetes mellitus, gestational diabetes mellitus, infertility drug treatment and assisted reproductive techniques, history of neonatal death, preterm birth, and congenital malformations all significantly increased the risk of perinatal death. Ethnic minority, income > 5,000, multiparous women, and cesarean section associated with reduced risk of perinatal death.

**Conclusion:**

Some factors of maternal sociodemographic characteristics, abnormal pregnancy history, lifestyle and habits during early pregnancy, and complications of pregnancy were associated with the risk of perinatal death.

## Introduction

Perinatal death, defined as a composite of stillbirths (fetal death at or after 28 weeks of gestation) and early neonatal deaths (neonatal death within 7 days of live birth), remains a major health problem globally (1, 2). It is estimated that there were 1.8 million early neonatal deaths and 2 million stillbirths in 2019 (3). The perinatal death rate has declined over the past 50 years (4). Although the stillbirth rate has been relatively stable, the rate of stillbirth is 10 times higher in developing countries compared to developed countries (5). Although neonatal deaths have been steadily decreasing, increased efforts to improve progress are still needed. It is estimated that 27.8 million neonatal deaths will occur from 2018 to 2030 if there are no decrements in neonatal mortality (6). Ninety-eight percent of globally reported perinatal deaths occur in developing countries (7), and there is a need to focus on reducing perinatal mortality in developing countries. The rate of stillbirth dropped by 4–6% from 2000 to 2015 in China. However, the rate of neonatal death is consistently higher than the rate of under-five death, and the decrement of stillbirth rate is slower than that of other adverse maternal and infant outcomes (8).

The effects of perinatal death are so devastating for mothers and their families, with long-term economic, psychological, and social consequences (9–11). Despite the slow progress in preventing perinatal deaths, ending preventable perinatal death remains a high concern for international public health (12). Numerous factors have been associated with perinatal death. Identifying risk factors and protective factors can facilitate the development of preventive strategies and improve clinical outcomes. Socioeconomic and demographic factors such as maternal age, education, and wealth are often associated with perinatal mortality (13). Some studies suggest parental health-related behaviors such as smoking and alcohol consumption are associated with stillbirths (14, 15). In addition, pregnancy complications such as gestational diabetes mellitus and gestational hypertension can increase the risk of perinatal death (16). Therefore, the use of antenatal interventions, modification of risk factors, and management of pregnancy complications can be taken into account to prevent perinatal death.

No comprehensive perspective investigation about the association of determinants with perinatal death in South China was conducted before. In order to recognize current determinants of perinatal death so as to guide future preventative care efforts, this study is based on a prospective cohort study to investigate the association of maternal and fetal characteristics with the risk of perinatal mortality and provide more evidence for protective factors and risk factors of perinatal death.

## Materials and Methods

### Recruitment of Study Participants

A prospective cohort study was conducted in Hunan provincial Maternal and Child Health Care Hospital in China. From March 13, 2013, to December 31, 2019, pregnant women who received their first antenatal care between 8 and 14 weeks of gestation were approached and invited to join the cohort. The study included pregnant women who met the inclusion criteria. The inclusion criteria for participation included being 18 years or older, intending to receive prenatal care and to deliver at the study hospital, and provided informed consent to be in the evaluation. Participants who met any of the following criteria were excluded from this study: (i) unable to cooperate with the investigation due to mental illness or extreme emotional instability; (ii) did not provide a blood sample or did not complete the questionnaire; (iii) women whose child had any known chromosomal abnormalities or syndromic CHD; and (iv) multiple pregnancies. Our study was approved by the ethics committee of the Xiangya School of Public Health of Central South University, and written informed consent was obtained from all mothers.

### Data Collection

The trained investigators used a self-designed questionnaire to collect the following information through one-to-one interviews, including sociodemographic characteristics (i.e., maternal obesity, ethnicity, and occupation); maternal history (i.e., adverse pregnancy, gestational diabetes mellitus, cesarean section, and neonatal death), lifestyle and habits during early pregnancy (i.e., drinking, smoking, and taking antidepressants); complications of pregnancy (i.e., gestational diabetes mellitus and gestational hypertension); and folate supplementation before or during this pregnancy. All participants were followed up until 3 months after birth to collect perinatal survival status and pregnancy outcomes (i.e., cesarean section, perinatal death, preterm birth, and congenital malformations). Data on disease diagnosis were confirmed by medical records.

### Definition and Assessment on Variables

The principal outcome of the study was perinatal death. We defined perinatal death as a death of offspring occurring at or after 28 weeks of gestation or up to 7 days after birth (17). The perinatal mortality rate was defined as the number of perinatal deaths per 1,000 births (including live births and stillbirths) in this study. Maternal education was categorized as ≤ 9 and > 9 years. Maternal ages were grouped as < 25, between 25 and 29, and ≥ 30 years. Residence was categorized as urban and rural. Ethnicity was categorized as Han nationality and other. Occupation was regrouped as unemployment and employment. Income was categorized as ≤ 5,000 and > 5,000 (RMB). Parity was defined as the number of deliveries a woman had, which was divided into nullipara (zero delivery) and multipara (one or more times deliveries). Mode of conception was categorized as spontaneous conception, spontaneous conception after infertility drug treatment (ovulation induction and simple drug therapy), and conception after assisted reproductive technology treatment [*in vitro* fertilization (IVF) and intracytoplasmic sperm injection (ICSI)]. Obesity was defined as a body mass index (BMI) > 30 kg/m^2^. Preterm births were defined as infants born less than 37 weeks of gestation. Congenital malformation was defined as perinatal abnormalities (including live births and stillbirths), which were probably of prenatal origin, including genetic defects and chromosomal abnormalities. Results of other variables were divided into yes or no, with yes indicating the occurrence of these outcomes.

### Statistical Analysis

Categorical variables were presented as rates or percentages. Generalized linear models of the Poisson family, with a log link, were utilized to calculate the relative risk (RR) and 95% CI for perinatal death in offspring associated with determinants. Unadjusted RR was calculated by univariate Poisson regression. Adjusted RR (aRR) was calculated by multivariate Poisson regression to control for potential confounders. Initially, all independent variables about perinatal mortality were included in the crude analyses. Only variables associated with perinatal death (*p* < 0.05) in the univariable analysis were entered into multivariate regression analyses. The database was developed using EpiData version 3.1 software. All analyses were performed using R software, version 3.6.1 (R Foundation for Statistical Computing).

## Results

**FIGURE 1:**
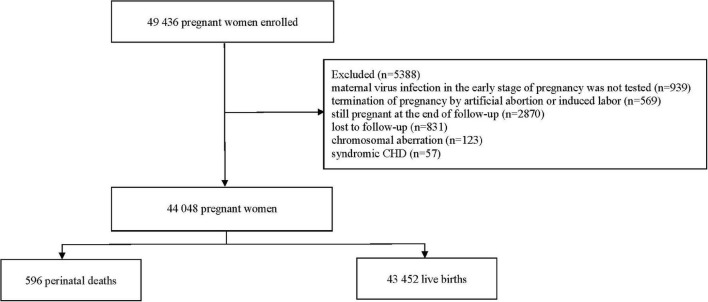
Flowchart of the study.

From March 13, 2013, to December 31, 2019, a total of 49,436 pregnant women with singleton pregnancies were registered and enrolled in the cohort during their first prenatal care in the early stage of pregnancy. According to the inclusion and exclusion criteria, the following pregnant women were excluded: (i) termination of pregnancy by artificial abortion or induced labor because of accidental pregnancy or ectopic pregnancy (*n* = 568; 1.1%); (ii) still pregnant at the end of follow-up (*n* = 2,870; 5.8%); (iii) lost to follow-up (*n* = 831; 1.7%); (iv) maternal virus infection in the early stage of pregnancy was not tested (*n* = 939; 1.9%); (v) their children were diagnosed with a chromosomal aberration (*n* = 123; 0.2%); and (vi) their children were diagnosed with syndromic CHD (*n* = 57; 0.1%). A total of 44,048 eligible pregnant women were included in the analysis (Figure 1). Of these, 43,452 (98.6%) were live births, and 596 (1.4%) were perinatal deaths. The perinatal mortality rate was 13.5 deaths per 1,000 births.

### Univariate Analysis

The sociodemographic characteristics of the study participants are presented in Table 1. The unadjusted analysis showed that maternal education level ≤ 9 years, living in rural areas, maternal obesity, and being employed were factors associated with increased risk of perinatal death. Ethnic minority and income > 5,000 were associated with decreased risk of perinatal death.

**TABLE 1:** Sociodemographic characteristics and unadjusted relative risk (RR) for perinatal death among 44,048 pregnant women.

Risk factors	Total (*n* = 44,048)	Perinatal death (*n* = 596)	Perinatal death risk/1,000 births	*p*	Unadjusted RR (95% CI)
**Maternal ages (years)**					
<25	4,980	50	10.0		1
25–29	19,724	336	17.0	**<0.001**	**1.70 (1.26–2.28)**
≥30	19,344	210	10.9	0.618	1.08 (0.80–1.47)
**Maternal education**					
>9 years	13,984	130	9.3		1
≤9 years	30,064	466	15.5	**<0.001**	**1.67 (1.37–2.02)**
**Residence**					
Urban	25,918	110	4.2		**1**
Rural	18,130	486	26.8	**<0.001**	**6.32 (5.14–7.76)**
**Ethnicity**					
Han	41,818	586	14.0		**1**
Other	2,230	10	4.5	**<0.001**	**0.32 (0.17–0.60)**
**Maternal obesity**					
No	43,590	582	13.4		**1**
Yes	458	14	30.6	**<0.001**	**2.29 (1.36–3.86)**
**Occupation**					
Unemployment	14,846	76	5.1		**1**
Employment	29,202	520	17.8	**<0.001**	**3.52 (2.77–4.49)**
**Income**					
≤5,000	30,902	532	17.2		**1**
>5,000	13,146	64	4.9	**<0.001**	**0.28 (0.22–0.37)**

*Bold values indicate a statistically significant difference between two groups (P < 0.05).*

The findings of the maternal history associated with perinatal death are shown in Table 2. The unadjusted analysis showed that history of gestational hypertension, history of neonatal death, history of gestational diabetes mellitus, infertility drug treatment, and assisted reproductive techniques were associated with increased risk of perinatal death. Multiparous women and a history of cesarean section were associated with decreased risk of perinatal death.

**TABLE 2:** Maternal history and unadjusted relative risk (RR) for perinatal death among 44,048 pregnant women.

Risk factors	Total (*n* = 44,048)	Perinatal death (*n* = 596)	Perinatal death risk/1,000 births	*p*	Unadjusted RR (95% CI)
**Parity**					
Primiparous	19,562	398	20.3		1
Multiparous	24,486	198	8.1	**<0.001**	**0.40 (0.34–0.47)**
**History of abortion**					
No	40,100	540	13.4		1
Yes	3,948	56	14.2	0.709	1.50 (0.80–1.38)
**History of stillbirth**					
No	43,224	586	13.6		1
Yes	824	10	12.1	0.727	0.90 (0.48–1.67)
**History of intrauterine embryo arrest**					
No	42,596	572	13.4		1
Yes	1,452	24	16.5	0.315	1.23 (0.82–1.85)
**History of adverse pregnancy**					
No	37,160	494	13.3		1
Yes	6,888	102	14.8	0.318	1.11 (0.90–1.38)
**History of gestational diabetes mellitus**					
No	39,578	500	12.6		1
Yes	4,470	96	21.5	**<0.001**	**1.70 (1.36–2.11)**
**History of gestational hypertension**					
No	40,326	524	13.0		**1**
Yes	3,722	72	19.3	**0.001**	**1.49 (1.17–1.90)**
**History of neonatal mortality**					
No	43,548	582	13.4		**1**
Yes	500	14	28.0	**0.006**	**2.10 (1.22–3.53)**
**History of cesarean section**					
No	39,676	580	14.6		**1**
Yes	4,372	16	3.7	**<0.001**	**0.25 (0.15–0.41)**
**Mode of conception**					
Spontaneous conception	19,128	148	7.7		**1**
Infertility drug treatment	14,976	188	12.6	**<0.001**	**1.62 (1.310–2.01)**
Assisted reproductive techniques	9,944	260	26.1	**<0.001**	**3.38 (2.77–4.13)**

*Bold values indicate a statistically significant difference between two groups (P < 0.05).*

The unadjusted analysis on the pregnancy-related factors associated with perinatal death (Table 3) showed that taking antidepressants during early pregnancy was the factor associated with increased risk of perinatal death.

**TABLE 3:** Lifestyle and habits during early pregnancy and unadjusted relative risk (RR) for perinatal death among 44,048 pregnant women.

Risk factors	Total (*n* = 44,048)	Perinatal death (*n* = 596)	Perinatal death risk/1,000 births	*p*	Unadjusted RR (95% CI)
**Taking antidepressants during early pregnancy**					
No	42,978	570	13.3		1
Yes	1,070	26	24.3	**0.002**	**1.83 (1.24–2.70)**
**Folic acid consumption before or during pregnancy**					
No	42,072	564	13.4		1
Yes	1,976	32	16.2	0.294	1.21 (0.85–1.72)
**Colds during early pregnancy**					
No	40,005	539	13.5		1
Yes	4,043	57	14.1	0.743	1.05 (0.80–1.37)
**Smoking during early pregnancy**					
No	43,444	584	13.4		1
Yes	604	12	19.9	0.176	1.48 (0.84–2.60)
**Exposure to secondhand smoke during early pregnancy**					
No	40,900	564	13.8		1
Yes	3,148	32	10.2	0.092	0.74 (0.52–1.05)
**Alcohol consumption during early pregnancy**					
No	43,484	592	13.6		1
Yes	564	4	7.1	0.192	0.52 (0.20–1.39)

*Bold values indicate a statistically significant difference between two groups (P < 0.05).*

The unadjusted analysis on the complications and outcomes of pregnancy-associated with perinatal death (Table 4) found that gestational diabetes mellitus, preterm birth, and congenital malformations were factors associated with increased risk of perinatal death. Cesarean section was associated with decreased risk of perinatal death.

**TABLE 4:** Complications and outcomes of pregnancy and unadjusted relative risk (RR) for perinatal death among 44,048 pregnant women.

Risk factors	Total (*n* = 44,048)	Perinatal death (*n* = 596)	Perinatal death risk/1,000 births	*p*	Unadjusted RR (95% CI)
**Gestational diabetes mellitus**					
No	39,762	496	12.5		1
Yes	4,286	100	23.3	**<0.001**	**1.87 (1.51–2.31)**
**Gestational hypertension**					
No	42,112	578	13.7		1
Yes	1,936	18	9.3	0.102	0.68 (0.43–1.08)
**Cesarean section**					
No	21,832	522	23.9		1
Yes	22,216	74	33.3	**<0.001**	**0.14 (0.11–0.18)**
**Preterm birth**					
No	36,754	382	10.4		**1**
Yes	7,294	214	29.3	**<0.001**	**2.82 (2.39–3.33)**
**Congenital malformations**					
No	41,768	445	10.7		**1**
Yes	2,280	151	66.2	**<0.001**	**6.22 (5.19–7.44)**

*Bold values indicate a statistically significant difference between two groups (P < 0.05).*

### Multivariable Analysis

**FIGURE 2:**
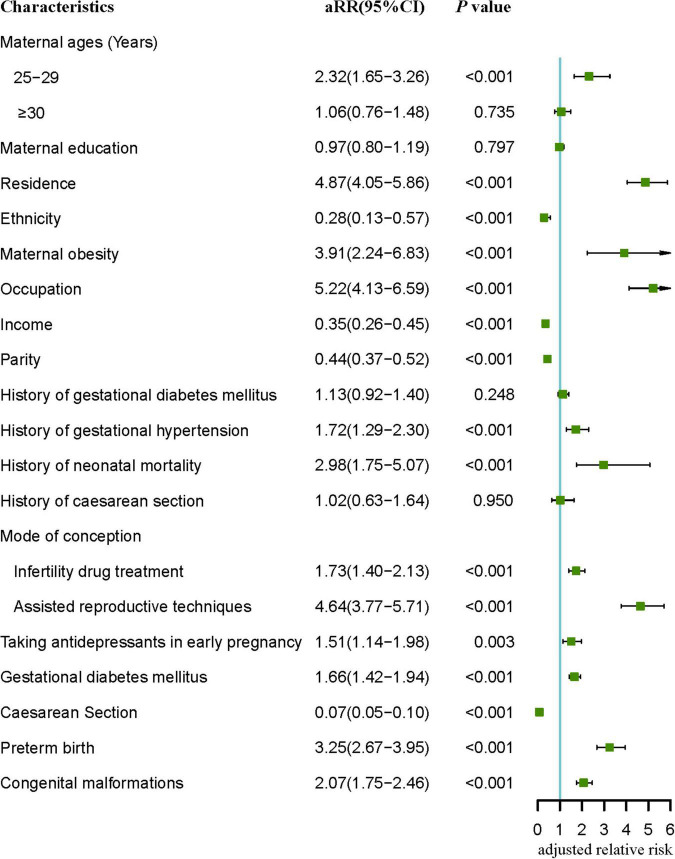
Multivariate analysis of significant (*p* < 0.05) risk factors in Tables 1–4. aRR, adjusted relative risk.

After adjustment, the results of the multivariate Poisson regression model (Figure 2) showed that maternal obesity (aRR = 3.91, 95% CI: 2.24–6.85), being employed (aRR = 5.22, 95% CI: 4.13–6.59), history of gestational diabetes mellitus (aRR = 1.13, 95% CI: 0.92–1.40), history of gestational hypertension (aRR = 1.72, 95% CI: 1.29–2.30), history of neonatal death (aRR = 2.98, 95% CI: 1.75–5.07), taking antidepressants during early pregnancy (aRR = 1.51, 95% CI: 1.14–1.94), gestational diabetes mellitus (aRR = 1.66, 95% CI: 1.42–1.94), infertility drug treatment (aRR = 1.73, 95% CI: 1.40–2.13) and assisted reproductive techniques (aRR = 4.64, 95% CI: 3.77–5.71), preterm birth (aRR = 3.25, 95% CI: 2.67–3.95), and congenital malformations (aRR = 2.07, 95% CI: 1.75–2.46) were still associated with an increased risk of perinatal death. And ethnic minority (aRR = 0.28, 95% CI: 0.13–0.57), income > 5,000 (aRR = 0.35, 95% CI: 0.26–0.45), multiparous women (aRR = 0.44, 95% CI: 0.37–0.52), and cesarean section (aRR = 0.07, 95% CI: 0.05–0.10) were still associated with a decreased risk of perinatal death.

## Discussion

In this prospective cohort study, we exhaustively explored the association of maternal and fetal characteristics with perinatal death. Independent risk factors for perinatal death were maternal obesity, being employed, history of gestational hypertension, taking antidepressants during early pregnancy, history of gestational diabetes mellitus, gestational diabetes mellitus, infertility drug treatment and assisted reproductive techniques, history of neonatal death, preterm birth, and congenital malformations. Ethnic minority, income > 5,000, multiparous women, and cesarean section were the protective factors of perinatal death. The perinatal death rate in the present research was 13.5 per 1,000 births, which was higher than in developed countries such as the United States (6.0/1,000 births) (18) and Ireland (5.4/1,000 births) (19). But the perinatal mortality in this study was much lower than the 41.6 per 1,000 births reported in northern Tanzania (20) and 75.3 per 1,000 births reported in Eastern Sudan (21). These differences in the perinatal mortality were likely attributable to differences in population health, lifestyle and habits, and availability of prenatal and obstetric emergency care between developed and developing countries. The specific reasons for differences in perinatal mortality between developed and developing countries also require more studies.

Our study found that after controlling for other factors, the risk of perinatal death in the offspring of mothers aged 25–29 was higher than that of mothers aged < 25, and advanced maternal age was not associated with perinatal death. Usynina et al. (22) also found that there was no association of advanced maternal age with perinatal death, because the association between maternal age and adverse pregnancy outcomes might be affected by age-related confounders or intermediates (23). A case–control study indicated that the offspring of mothers with only primary education or no schooling had a 5.4-fold higher risk of perinatal death than educated mothers (24). This finding is in contradiction with a prospective study that suggested that the offspring of women who completed secondary education or above had a higher risk of perinatal death (25). In our study, maternal education of less than 9 years elevated the incidence of perinatal death in the univariate analysis. But it lost statistical significance after adjustment. The previous study found that perinatal mortality was higher in rural areas compared to urban areas (26). We found the same result that can be due to the remoteness and the short availability of the health service in rural areas. In addition, urban residents possessed better wealth and better medical care services for their newborns (27). Maternal employment might also have a negative impact on fetuses and newborns because mothers who are employed might not have enough time to rest and care for their babies. Our study suggested that maternal employment may be one of the risk factors of perinatal death, consistent with previous studies (28). Prior literature indicated that wealthy families were more likely to receive higher cash incomes than poor ones, thus enabling them to have access to a quality diet and better medical care (27). Our study found that income > 5,000 was a protective factor for perinatal death. The RR of perinatal mortality was 3.91 times in babies born to obese mothers compared with non-obese mothers. A number of studies had revealed similar results in both developed and developing countries (29–31). Obesity might affect placental function (32) or lead to other maternal obesity-related pregnancy complications (33). Obesity was still the dominant and modifiable risk factor for perinatal mortality. According to reports, effective weight loss during pregnancy can decrease the occurrence of gestational diabetes mellitus, which had a significant impact on the gestational age at birth and the rate of neonatal death (34).

The offspring of multiparous women had a lower risk of perinatal death as compared to those of nulliparous women. Our research was consistent with the studies conducted in Bangladesh, Uganda, and Burkina Faso (13, 35, 36). Neonatal asphyxia and perinatal death easily occurred in the offspring of nulliparous women (37), which might be because nulliparous women were prone to delivery complications, such as obstruction (38). One of the vital factors of perinatal death in this survey was the history of neonatal death. A study conducted in Missouri (United States) revealed that women with a history of neonatal death had an increased risk of subsequent stillbirths (39) because women with a history of neonatal death might suffer from anatomical problems associated with the pelvis, the uterus, or any parts of the birth canal, resulting in complications and perinatal mortality. This also demonstrated the significance of special investigation and care during the pregnancy and childbirth period to women who had a history of neonatal death in order to avoid subsequent adverse pregnancy outcomes. Elevated evidence verified that IVF/ICSI significantly increased the risk of adverse pregnancy outcomes in offspring as compared with spontaneous conception (40–42). Our study found that compared with that of spontaneous conception, the RR of perinatal mortality of IVF/ICSI and infertility drug treatment was 4.642 and 1.726, respectively. This might be due to the increased risk of systemic complications in pregnant women through assisted reproductive techniques, and the operation in the treatment may produce certain stimulation to the uterus. The use of ovulation induction drugs resulted in the body’s estrogen levels greatly exceeding the normal physiological level and increased the risk of perinatal death (43, 44). Antidepressants might have negative effects on people such as memory difficulties, irritability, reasoning loss, and coordination disorders (45). Our study found that taking antidepressants during early pregnancy increased the risk of perinatal mortality. As far as we know, this was the first evidence that the associations of taking antidepressants during early pregnancy with perinatal mortality were discovered through a prospective cohort study. However, the specific mechanism and whether there was a quantitative relationship between the two needs further research in the future.

A study of gestational hypertension suggested that pregnant women with gestational hypertension had a remarkably elevated risk of perinatal death as compared with pregnant women without gestational hypertension (46). However, our study found that gestational hypertension did not correlate with perinatal death, and a history of gestational hypertension was a risk factor for perinatal mortality. There had been controversy over whether maternal gestational diabetes had adverse effects on the perinatal mortality of offspring (16, 47, 48). Theoretically, the high maternal glucose levels in pregnant women with diabetes contributed to high fetal glucose levels. Fetal hyperglycemia consumed the circulating oxygen, thus leading to hypoxia and acidosis, and the maternal oxyhemoglobin reduced the release of oxygen to tissues, thus resulting in fetal chronic hypoxia (49). Our study prospectively confirmed that mothers with gestational diabetes mellitus had higher perinatal mortality. Interestingly, Feig et al. (50) revealed that women with gestational diabetes mellitus had a lower risk of perinatal death than non-diabetic women. They argued that stricter regulation in pregnancies of women with gestational diabetes mellitus contributed to the low perinatal death in offspring. This provided evidence and ideas for us to manage and control the perinatal mortality of pregnancies with gestational diabetes mellitus.

Although the individual research had found that cesarean section did not affect perinatal mortality compared with vaginal delivery (51), most studies showed the rate of perinatal death was significantly higher among infants who had undergone cesarean section (52, 53). The reason was that the indication for cesarean section was usually an emergency, so severe obstetric problems or an abnormal delivery resulted in an increased risk of fetal death (54). Our study found that cesarean section was a protective factor for perinatal mortality in accordance with the results of a recent retrospective cohort study (20). This might be related to the planned cesarean section in China, which was not carried out in an emergency condition. On the other hand, the provider’s good skills and timeliness of cesarean section were still associated with the decrease of perinatal mortality through cesarean section. Preterm birth might exacerbate the risk of perinatal mortality because premature infants had insufficient anatomical and physical development of all systems. More seriously, premature infants with respiratory distress syndrome, immature pulmonary development, and susceptibility to infections caused by an underdeveloped immune system were prone to death (55). Our survey suggested that the RR of perinatal mortality in preterm infants was 3.25 times higher than that in non-preterm infants.

This study utilized a large sample of pregnant women and is a prospective cohort study. This gave us a powerful ability to detect the association between interesting exposure and perinatal death. Many findings were discovered in this study in accordance with the previous studies, which provide further confidence for our analysis. However, some limitations should be considered.

First, the study population might conceal their risk behaviors, such as history of smoking and history of drinking, thus underestimating the association between these behaviors and perinatal adverse outcomes. Second, the data of this study were mainly from the same hospital, and the sample source may be concentrated on a certain type of population, which may affect the representativeness of the sample and lead to selection bias. Third, although adjusting for a variety of potential confounding factors, we still cannot completely exclude the involvement of the possibility of residual confounding, because these factors investigated in this research did not account for all the determinants of perinatal death. Fourth, our study was limited by our inability to distinguish antepartum from intrapartum deaths and the likely misclassification of some early neonatal deaths as stillbirths, so the perinatal deaths were not separated into stillbirths and early neonatal deaths to analysis in our research. In addition, the causes of perinatal death were not involved in our study.

## Conclusion

The present study is the first to comprehensively evaluate the association of maternal and fetal characteristics with the risk of perinatal death, which suggests that some factors such as maternal sociodemographic characteristics, abnormal pregnancy history, lifestyle and habits during early pregnancy, and complications of pregnancy are significantly associated with perinatal death. To identify some modifiable risk factors and to give effective reduction and control are an important link to reducing perinatal mortality. However, the limitations in this study should be carefully taken into account. In the future, more specific studies are required to refine and confirm our findings.

## Data Availability Statement

The raw data supporting the conclusions of this article will be made available by the authors, without undue reservation.

## Ethics Statement

The studies involving human participants were reviewed and approved by Ethics Committee of the Xiangya School of Public Health of Central South University. The patients/participants provided their written informed consent to participate in this study.

## Author Contributions

YpL: study design, statistical analysis, and manuscript writing. JQ and TY: manuscript revision and review. YpL, QL, TW, LC, SZ, YhL, JD, JL, MS, JW, XS, and JS: data collection. All authors contributed to the article and approved the submitted version.

## Conflict of Interest

The authors declare that the research was conducted in the absence of any commercial or financial relationships that could be construed as a potential conflict of interest.

## Publisher’s Note

All claims expressed in this article are solely those of the authors and do not necessarily represent those of their affiliated organizations, or those of the publisher, the editors and the reviewers. Any product that may be evaluated in this article, or claim that may be made by its manufacturer, is not guaranteed or endorsed by the publisher.
